# E6/E7 Sequence Diversity of High-Risk Human Papillomaviruses in Two Geographically Isolated Populations of French Guiana

**DOI:** 10.3390/microorganisms8111842

**Published:** 2020-11-23

**Authors:** Mathieu Nacher, Gersande Godefroy, Valentin Dufit, Maylis Douine, Fatiha Najioullah, Raymond Césaire, Nadia Thomas, Kinan Drak Alsibai, Antoine Adenis, Vincent Lacoste

**Affiliations:** 1 Centre d’Investigation Clinique—CIC INSERM 1424, Centre Hospitalier de Cayenne “Andrée Rosemon”, CEDEX 97306 Cayenne, France; valentin.dufit@ch-cayenne.fr (V.D.); maylis.douine@ch-cayenne.fr (M.D.); antoine.adenis@ch-cayenne.fr (A.A.); 2 Département Formation Recherche Santé, Université de Guyane, Cayenne, CEDEX 97300 Guyane, France; 3 Laboratoire des Interactions Virus-Hôtes, Institut Pasteur de la Guyane, CEDEX 97306 Cayenne, France; gersande.godefroy@wanadoo.fr (G.G.); vincent.lacoste@pasteur.fr (V.L.); 4 Service de Virologie, Martinique University Hospital, Fort-de-France, CEDEX EA, 7524 Martinique, France; fatiha.najioullah@chu-martinique.fr (F.N.); raymond.cesaire@chu-martinique.fr (R.C.); 5 Centres Délocalisés de Prévention et de Soins, Centre Hospitalier de Cayenne “Andrée Rosemon”, CEDEX 97306 Cayenne, France; nf.thomas@wanadoo.fr; 6 Service d’Anatomopathologie, Centre Hospitalier de Cayenne “Andrée Rosemon”, CEDEX 97306 Cayenne, France; Kdrak.alsibai@doctor.com; 7 Département de Virologie, Institut Pasteur, CEDEX 75015 Paris, France

**Keywords:** human papillomavirus, genotype, variant, remote populations, Maroni, Oyapock

## Abstract

Amerindian and Maroon populations of French Guiana have been living in isolation for generations and sexual networks remained mostly endogamous. The present study aimed to describe the phylogeny of E6 and E7 sequences of the most common high-risk HPV genotypes in these regions, to ascertain the diversity of intra-type variants and describe evolutionary relationships. There were 106 women with at least one of HPV16, 18, 31, 52, 58, and 68 genotypes. The most clear-cut phylogenetic pattern was obtained for HPV18 and HPV58 for which the major branches were crisply divided between Amerindian villages on the Oyapock and Maroon villages on the Maroni. Such clustering was less clear for HPV31 and 52. For HPV16, there was also some evidence of clustering on the Oyapock with type A European viruses and on the Maroni with type B and C African viruses among Maroon women. HPV68 showed the largest sequence heterogeneity of the six genotypes at both nucleotide and amino acid levels and was restricted to Maroon women. The present results show that there were significant geographically based differences of E6 and E7 oncogenes. These differences were compatible with different ancestral virus populations and local virus evolution in a context of prolonged population isolation.

## 1. Introduction

In French Guiana, cervical cancer represents the second most frequent cancer in females with a standardized incidence rate of 30.3 per 100,000 women. It is also a substantial cause of mortality [1,2]. French Guiana is mostly covered by primary forest, the population density is low, and 20% are living in remote parts of the territory only accessible by canoe. There is a network of health centers connected to the main hospital but accessing care can be difficult when persons live outside of the main villages. Despite the absence of a significant difference in incidence between rural and urban areas, there are differences in diagnostic delays, hence 77% of women in the rural areas have lesions that metastasize beyond the cervix, whereas this is the case for only 44% of women living in urban areas [3]. Health professionals usually consider that because of the early sex initiation, and perhaps due to cultural practices of vaginal steam baths and dry sex, populations living in these remote parts are at greater risk of cervical lesions due to HPV [4]. In fact, this applies for all sexually transmitted agents, notably HIV and HTLV1 [5]. HPV16 and HPV18 are involved in nearly three-quarters of cervical cancers in Europe, and thus vaccination against these genotypes is now recommended in France. Information on HPV genotypes in cancer samples is not yet available in French Guiana. However, we have shown that among women living on the Maroni and Oyapock rivers, the prevalence of HPV was particularly high and that the genotypes found by performing HPV testing were quite different and justified the use of nonavalent vaccines to prevent cervical cancer in these remote areas of high prevalence [3,6,7]. Moreover, the proportion of abnormal smears was higher than what is observed in mainland France. Hence, 10% of women had cytological anomalies, which is twice what was observed in the Amazonas state in Brazil [8]. Among those with an interpretable smear, 1.2% had HSIL, which was 4 times the proportion observed in the greater Paris region in 2002 [9]. The Amerindians and Maroons living along the Oyapock and Maroni rivers have been living along these rivers for generations, at least since the 19th century for Maroons, and much more for Amerindians. They have singular epidemiological features due to endogamous sexual networks and different cultural practices [10]. There may also be differences in terms of genetic predispositions to viral infections, and specific viral variants may lead to greater viral persistence and oncogenicity [11,12,13,14]. Indeed, studies in the Brazilian Amazon have shown the presence of such variants. The role of HPV in cervical cancer is largely mediated through *E6* and *E7* genes. E7 is an oncoprotein that immortalizes epithelial cells with the cooperation of the E6 oncoprotein [15]. Overexpression of these proteins leads to changes in cellular pathways and functions leading to malignant transformation [16]. Between 2012 and 2014, an HPV prevalence study was conducted in women aged 20–65 years living in these remote parts of French Guiana. HPV16, 18, 31, 52, 58, and 68 were the most frequent high-risk genotypes among these women [3,6]. The objective of the present study was to describe the phylogeny of E6 and E7 sequences of the most common high-risk HPV genotypes in these regions. More specifically, because of the differences in terms of sexual network, ethnicity, history, and geographical isolation, we aimed to test the hypothesis that E6 and E7 intra-type variant sequences were phylogenetically different, giving perhaps further credence to the hypothesis that variants found in Amerindians were indeed more oncogenic.

## 2. Materials and Methods

### 2.1. Study Conduct

The study was conducted between December 2012 and September 2014 among women aged 20–65 years, sexually active and living in the remote villages on the Maroni and Oyapock rivers. In all villages, communication about HPV, cervical cancer, and the project sensitized the local populations before beginning the inclusion of patients. Administrative and traditional local authorities and health workers were also informed. Information on the dates of the study in the village were given by radio messages. Women who wanted to be screened came to the health center on the day the mission arrived. After giving informed consent, samples were taken and questionnaires were filled. Socioeconomic and demographic data as well as gynecological and obstetrical history were collected with a short-standardized questionnaire. Exclusion criteria were hysterectomy and pregnancy >3 months.

### 2.2. Sample Collection

Samples collected on the Thin-Prep liquid medium were kept in a cooler until the end of the mission. The samples were then flown to Martinique, to the virology and pathology laboratories of Fort-de-France Hospital, and to the Institut Pasteur de la Guyane for cytological and molecular analyses [3]. HPV testing and genotyping has already been done using the Greiner Bio-One kit that allows the identification of 25 different HPV types, of which 18 are high-risk, as well as the identification of multiple infections [3]. When HPV was positive and cytology negative, a gynecological follow-up was recommended to make sure that HPV positivity disappeared or that cytological lesions did not appear.

### 2.3. Type-Specific E6 and E7 Gene Amplification

Total DNA was extracted from a minimum of 2 mL of sample in liquid phase using a NucliSens^®®^ easyMAG^TM^ bio-robot (bioMérieux, Marcy-l'Étoile, France). To verify the DNA integrity of each sample, primers for human *β-globin*, PCO4 (5′CAACTTCATCCACGTTCACC3′) and GH20 (5′GAAGAGCCAAGGACAGGTAC3′), were first used. E6 and E7 type-specific primers were designed using the MacVector 6.0 software (Oxford Molecular Ltd., Cambridgeshire, UK) and are described in Supplementary Table S1. Forward and reverse primers were designed upstream of E6 and downstream of E7, respectively, to amplify the full-length coding sequences of the two genes. Each PCR reaction was performed in a final volume of 50 µl using the AmpliTaq Gold DNA polymerase kit (Thermo Fischer Scientific, Waltham, MA, USA) on a GeneAmp 9700 PCR thermal cycler (Applied Biosystems, Foster City, CA, USA). PCR cycling conditions were 10 min at 94 °C, followed by 35 cycles of 30 sec at 94 °C, 30 s at 47–56 °C, 30 s at 72 °C, and a final extension of 10 min at 72 °C. The optimal annealing temperature was specific to each couple of primers (Supplementary Table S1). PCR products were visualized by electrophoresis on ethidium bromide-stained gels. When detected, amplicons of the predicted size were sent for sequencing to Genewiz^®®^, Takeley, UK (https://www.genewiz.com/). Amplicons were fully sequenced on both strands using the PCR primers.

### 2.4. Phylogenetic Analysis

Raw sequences were analyzed and edited in MEGA (v5.05) [17]. Sequence homology analyses were performed using the BLAST program at the National Center of Biotechnology Information (NCBI) (http://blast.ncbi.nlm.nih.gov/Blast.cgi). Then, multiple-sequence alignments with other previously published sequences from the same HPV genotype were constructed using the ClustalW program, and the alignments were checked manually. The sequences were translated into amino acids, and both nucleotide and amino acid sequences were checked for irregularities. Pairwise sequence identity (at the nucleotide and amino acid levels) of the concatenated E6 and E7 coding sequences was calculated with MEGA v5.05 using uncorrected *p*-distances. Phylogenetic trees were inferred from the aligned nucleotide sequences by using a maximum likelihood phylogenetic approach. The best-fitted model of nucleotide substitution was determined for each dataset using MEGA v5.05 under corrected Akaike information criteria (AICc). The Hasegawa-Kishino-Yano (HKY) model using a Gamma distribution (+G) with five rate categories (for HPV16, 18, 31, 52) or the HKY+G with (I) proportion of invariable sites (HPV58, 68), were identified as the optimal models of nucleotide evolution. One thousand bootstrap replicates were generated. Trees obtained were visualized using the FigTree v1.4 program (http://tree.bio.ed.ac.uk/software/figtree/). Information relative to the sets of representative sequences of each lineage and sub-lineage for each HPV type is available in Supplementary Table S2.

### 2.5. Data Collection and Analysis

The data were analyzed using MEGA v5.05 software (Pennsylvania State University, University Park, PA, USA) and Stata 13.0^®^ (STATA Corporation, College, Station, TX, USA) [17]. Unique sample identifiers included a three-letter code corresponding to the town of residence, which allowed one to determine if the sample came from the Oyapock or the Maroni rivers and if it came from an Amerindian or a Maroon village, followed by an Arabic numeral. French authorities do not authorize the collection of self-reported ethnicity. Therefore, in order to approach the origin country of origin, village of origin (there are known Maroon villages and Amerindian villages), and maternal language are used to differentiate ethnicities. The country of birth and maternal language allowed checking for discordances in genetic distance. For different lineages, crosstabulations were performed with river basins and statistical tests were performed (chi2 or Fisher’s tests).

### 2.6. Ethical and Regulatory Aspects

Regulatory and ethical approval was given on 13 March 2012 by the Comité d’Evaluation Ethique de l’Inserm (CEEI), approval n° 12-064, the Comité de Recherche Clinique (CoRC) Pasteur Institute on 19 April 2012, n° 2012-15, the Comité Consultatif sur le Traitement de l’Information en matière de Recherche dans le domaine de la Santé (CCTIRS) on 13 June 2012, n° 12-310, and the Commission Nationale de l’Informatique et des Libertés (CNIL) on 12 October 2012, n° 912459. All study participants gave written informed consent for the study and the publication of its results.

## 3. Results

As previously reported, 601 women with a satisfactory quality of cervical smear were included in the study [3,6,7]. Of the 540 women with normal cervical smears, 147 (27%) had a positive HPV test, whereas of the 61 women with abnormal cervical smears, 52 (85%) had a positive HPV test. Cytological anomalies were 30 ASCUS (Atypical Squamous Cells of Unknown Significance), 7 ASC-H (Atypical Squamous Cell evocating High grade lesion), 18 LSIL (Low-grade squamous intraepithelial lesion), 7 HSIL (High-grade Squamous Intraepithelial Lesion), and 3 glandular anomalies. HPV 52 was involved in the highest absolute number of abnormal cytology results (12/27). In proportion, it was similar to HPV 16 (8/18). By contrast, HPV 68 was frequent but rarely associated with cytological anomalies (3/20). This was statistically significant when compared with HPV 16 (*p* = 0.046) or 52 (*p* = 0.03).

Among the 199 that had a positive HPV test, 106 were infected or co-infected by one of the most frequent HR-HPV genotypes (HPV16, 18, 31, 52, 58, and 68). Ninety-two were mono-infected, 12 were co-infected, and 2 were infected by three of these genotypes (Table 1 and Table 2). 

Forty-three women originated from the different villages of the Oyapock (Table 1) and 63 from the Maroni (Table 2). Whereas HPV52 was the most widely geographically distributed HR genotype, found in eight of nine villages, followed by HPV16 and HPV31, detected in seven of the nine villages, the geographical distribution of HPV68 appeared to be almost exclusively restricted to the Maroni, with 18 of the 20 cases identified from four villages of the Maroni river (Table 2). Furthermore, HPV68 was also almost exclusively restricted to Maroons, whereas the other genotypes were identified in all ethnic groups (Table 1 and Table 2). In addition, looking at the three villages (Trois Sauts, Apatou, and Papaïchton) where the largest number of sequences were obtained (>19 sequences), the six distinct HPV genotypes were identified. Nevertheless, their proportion varied depending on the village. In particular, whereas HPV68 was the most predominant genotype in Apatou and Papaïchton, representing more than one-third of all sequences, it was the least present in Trois Sauts (3.2%) where the proportion of the five other genotypes varied from 12.9% to 25.8%. In these three villages, the proportion of HPV16 and HPV18 did not exceed 21% of sequences.

To further characterize the intra-type variants of these genotypes and their diversity, all samples were tested for E6/E7 genetic amplification and sequencing. Beta-globin PCR first confirmed the integrity of all the studied DNA samples. Then, use of different combinations of type-specific E6/E7 primers allowed obtaining 114 PCR products over the 122 expected. We did not get any amplification products for five mono-infected samples previously identified as positive for HPV16 (for one of them), HPV52 (one), HPV58 (two), and HPV68 (one) on the basis of the Greiner Bio-One kit, whereas for three dually-infected samples only one of the two genotypes was amplified (Table 1 and Table 2) [3]. Amplification products were thus obtained from 66 women with a normal cytology and from 35 with an abnormal cytology (Table 1 and Table 2). The complete coding sequences of the *E6* and *E7* genes were obtained for all of them. This allowed constructing multiple sequence alignments and performing phylogenetic analyses for each of these genotypes with previously published sequences extracted from GenBank (http://www.ncbi.nlm.nih.gov/nucleotide) belonging to the same genotype and representative of the main branches in order to assign the variants of our cohort to previously established intra-type clades [18,19].

### 3.1. Genetic Diversity and Phylogeny of HPV16 Sequences

With 18 samples, 11 from the Maroni and 7 from the Oyapock, HPV16 was the fourth most common HPV type in this cohort (Table 1 and Table 2). Nine sequences were identified from Native American-speaking women, five from Bushinengue-speaking women, and four from Portuguese-speaking women. The E6 sequences presented among themselves nine nucleotide substitutions of which five nonsynonymous associated with four amino acid changes at positions 10, 14, 78, and 83 (numbers here and thereafter refer to amino acid positions relative to the first methionine of the protein of interest), whereas E7 sequences presented four substitutions of which two non-synonymous at positions 29 and 63, N29S and S63Y (amino acid changes are based on HPV16 reference sequence, GenBank accession number K02718). So, the new sequences exhibited among themselves from 98.4% to 100% nucleotide identity and from 98% to 100% amino acid identity. They showed 98.3%–100% nucleotide identity and 97.6%–100% amino acid identity with the representative sequences of the different HPV16 lineages and sublineages.

Based on E6 protein sequences, the 18 isolates grouped into four distinct variants corresponding to the reference sequence as well as to variants L83V, R10T/Q14D/H78Y, and R10I/Q14D/H78Y (Supplementary Table S3) [20]. These variants were represented by eight, five, three and two sequences, respectively. Those corresponding to the reference and L83V sequences only differed from each other by one nonsynonymous substitution in *E6* located at nucleotide position 350 (G350T). According to the proposed nomenclature of Huertas-Salgado et al., isolates with the reference and L83V sequences belonged to the European branch (classes T350 and G350, respectively), whereas variants R10T/Q14D/H78Y and R10I/Q14D/H78Y belonged to the African 1 and 2 branches, respectively [20]. In addition, the two sequences (CAP31 and CGS57 isolates) of variant R10I/Q14D/H78Y presented the N29S amino acid change in E7 [17]. The three R10T/Q14D/H78Y sequences differed from each other by one nonsynonymous nucleotide substitution located at position 190 (C190A) of *E7* gene leading to S63Y mutation (PAN12 isolate). All other sequences had no amino acid change on E7 relative to the reference sequence.

**Figure 1:**
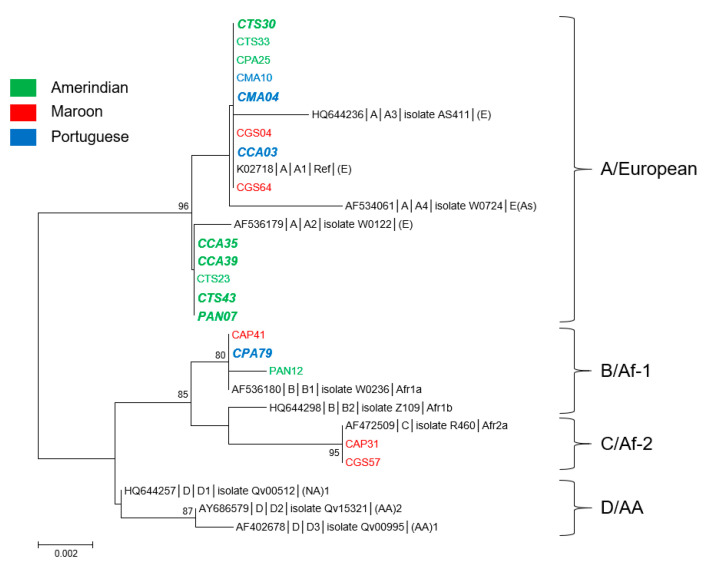
Phylogenetic tree of HPV16 variants from French Guiana. Maximum likelihood phylogenetic tree of HPV16 variants based on 747 bp of E6 and E7 concatenated sequences. Four clusters were identified as lineages A, B, C, and D. Only bootstrap values above 70% are represented in the branches. The tree is drawn to scale with branch lengths measured in the number of substitutions per site. Newly generated sequences, here identified by their IDs, are color-coded according to the mother tongue of the women from whom they were obtained. Oversized sequences in bold and in italics are those obtained from women with an abnormal cytology (either Atypical Squamous Cells of Unknown Significance (ASCUS), Atypical Squamous Cells, cannot exclude high grade lesion (ASC-H), Low Grade Squamous Intraepithelial Lesion (LSIL), High Grade Squamous Intraepithelial Lesion (HSIL), or glandular anomalies).

From a phylogenetic viewpoint, the 18 sequences fell into three well-supported clades corresponding to lineage A for 13 of them, to lineage B for 3, and to lineage C for 2 (Figure 1). All sequences from the Oyapock basin were of lineage A, whereas those from the Maroni basin were distributed among the three distinct lineages (Supplementary Table S3). In addition, eight of the nine sequences obtained from women of Amerindian mother tongue belonged to lineage A. The two lineage C sequences were identified from Maroon women, whereas the three B sequences were each identified from an Amerindian (PAN12), a Maroon (CAP41), and a Brazilian (CPA79) woman. Furthermore, of the 18 HPV16 sequences obtained, eight originated from women presenting cytological anomalies (Supplementary Table S3). The cross tabulations of lineages and river basins suggested patterns but failed to reach statistical significance (*p* = 0.11). None of the five sequences identified from women of Bushinengue language were associated with cytological anomalies, whereas five of the nine sequences from Native American-speaking women and three of the four from Portuguese-speaking women were associated with cytological anomalies. It is noteworthy that of the five sequences from Native American-speaking women presenting cytological abnormalities, four clustered together with the representative strain of A2 sublineage (Figure 1).

### 3.2. Genetic Diversity and Phylogeny of HPV18 Sequences

Fifteen HPV18 sequences were obtained, seven from women from the Oyapock basin and eight from the Maroni basin (Table 1 and Table 2).

The E6 sequences presented among themselves 14 nucleotide substitutions of which three non-synonymous associated with three amino acid changes at positions 15, 80, and 129. E7 sequences presented seven substitutions of which four nonsynonymous leading to three amino acid changes at positions 2, 71, and 92. Pairwise sequence comparison of E6/E7 concatenated sequences demonstrated that the new sequences exhibited among themselves from 98.1% to 100% nucleotide and amino acid identities. Comparison with representative sequences of the different HPV18 lineages showed from 98% to 100% nucleotide identity and from 97.7% to 100% amino acid identity.

**Figure 2:**
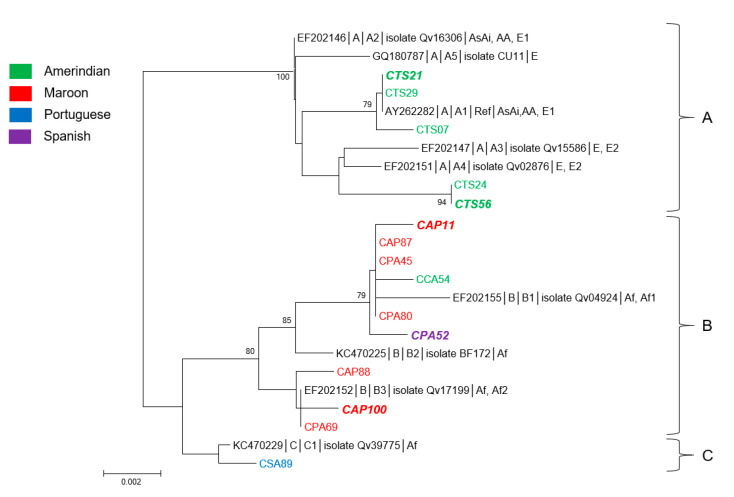
Phylogenetic tree of HPV18 variants from French Guiana. Maximum likelihood phylogenetic tree of HPV18 variants based on 789 bp of E6 and E7 concatenated sequences. Three clusters were identified as lineages A, B, and C. Only bootstrap values above 70% are represented in the branches. The tree is drawn to scale with branch lengths measured in the number of substitutions per site. Newly generated sequences, here identified by their IDs, are color-coded according to the mother tongue of the women from whom they were obtained. Oversized sequences in bold and in italics are those obtained from women with an abnormal cytology (either ASCUS, ASC-H, LSIL, HSIL, or glandular anomalies).

Phylogenetically, the sequences here identified were divided into three distinct lineages A, B, and C (Figure 2). All but one Amerindian women from the Oyapock basin were infected with a virus belonging to the lineage A, whereas all Maroon women were infected with a virus of lineage B (Supplementary Table S4). Despite the limited number of observations, the cross tabulations of lineages and river basins showed these patterns were statistically significant (*p* = 0.001). The only Amerindian woman who was not infected with a virus of lineage A was infected with a lineage B virus. She was from the village of Camopi, whereas all others were from Trois Sauts. The unique lineage C sequence was identified from a Brazilian woman. One-third of the sequences were identified from women with abnormal cytologies: two from Native American-speaking women from Trois Sauts, two from Bushinengue-speaking women, and one from a Spanish-speaking woman (Figure 2).

### 3.3. Genetic Diversity and Phylogeny of HPV31 Sequences

HPV31 was the third most prevalent HPV type of the study population. The complete E6 and E7 regions of 19 isolates were sequenced (Supplementary Table S5). A total of 18 nucleotide positions over 741 showed variations compared with the prototype sequence (accession number # J04353). Half of them were nonsynonymous, leading to nine amino acid substitutions, six located in E6 (at positions 52, 60, 64, 86, 123, and 138) and three in E7 (at positions 23, 46, and 62) sequences. The new sequences exhibited among themselves from 98.7% to 100% nucleotide identity and from 97.6% to 100% amino acid identity. They showed 98.5%–100% nucleotide identity and 97.2%–100% amino acid identity with the representative sequences of the different HPV31 lineages.

**Figure 3:**
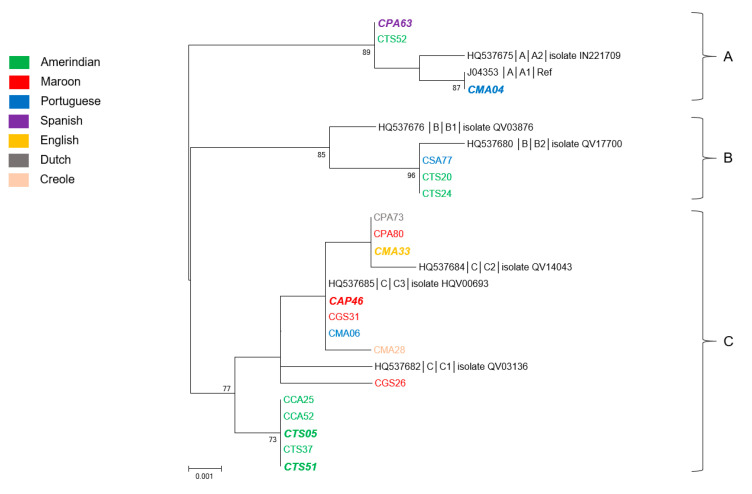
Phylogenetic tree of HPV31 variants from French Guiana. Maximum likelihood phylogenetic tree of HPV31 variants based on 741 bp of E6 and E7 concatenated sequences. Three clusters were identified as lineages A, B, and C. Only bootstrap values above 70% are represented in the branches. The tree is drawn to scale with branch lengths measured in the number of substitutions per site. Newly generated sequences, here identified by their IDs, are color-coded according to the mother tongue of the women from whom they were obtained. Oversized sequences in bold and in italics are those obtained from women with an abnormal cytology (either ASCUS, ASC-H, LSIL, HSIL or, glandular anomalies).

As shown in Figure 3, the phylogenetic tree generated from the E6/E7 concatenated nucleotide sequences distributed the newly generated sequences within the three distinct lineages, designated A, B, and C. Although the eight sequences identified from women of Native American language were distributed into the three lineages, the four sequences from women of Bushinengue language all belonged to the C lineage (Supplementary Table S5). The three women born in Brazil were each infected with a virus belonging to one of the three lineages. Regarding the four last women for whom an HPV31 type was identified, all but one were infected with a lineage C virus. Each of these women had different geographical origins and mother tongues: CPA63 corresponded to a Dominican person, CGS26 was born in Suriname, CMA33 was from Guyana, and CMA28 was a Guianese Creole. Interestingly, the five lineage C sequences identified from women of Native American language of two distinct villages from the Oyapock grouped together in a distinct, well-supported subclade (Figure 3). These sequences were identical to each other and presented from 99.3% to 99.6% of nucleotide identity and from 98.4% to 99.2% of amino acid identity with all other lineage C sequences. At last, the six HPV31 sequences identified from women with cytological anomalies were not associated with a particular lineage or sublineage but were divided among the three distinct lineages and were from women of different origins (Supplementary Table S5). The cross tabulations of lineages and river basins failed to reach statistical significance (*p* = 0.13).

### 3.4. Genetic Diversity and Phylogeny of HPV52 Sequences

With 27 sequences obtained from women from eight villages, 14 from the Oyapock, and 13 from the Maroni, HPV52 was the most common HPV type of the study population (Table 1 and Table 2). Thirteen sequences were obtained from women of Native American language from three villages of the Oyapock basin (11 sequences) and from two of the Maroni basin (two sequences), nine sequences from women from two villages from the Maroni having a Maroon language, and five from women from Saint Georges (Oyapock) and Maripasoula (Maroni) of Portuguese language (Supplementary Table S6).

Over 741 nucleotide positions, the E6/E7 sequences presented among themselves 24 nucleotide substitutions of which 13 nonsynonymous associated with 10 amino acid changes relative to the reference sequence (accession number X74481). The E6 coding sequences presented 3 nonsynonymous substitutions associated with 2 amino acid changes, whereas E7 sequences possessed 10 of them associated with 8 amino acid changes. The 27 new sequences presented among themselves from 98.1% to 100% nucleotide identity and from 96.8% to 100% amino acid identity. In addition, they showed from 98% to 100% and from 96.4% to 100% of nucleotide and amino acid identity, respectively, with the representative sequences of the different HPV52 lineages and sublineages.

**Figure 4:**
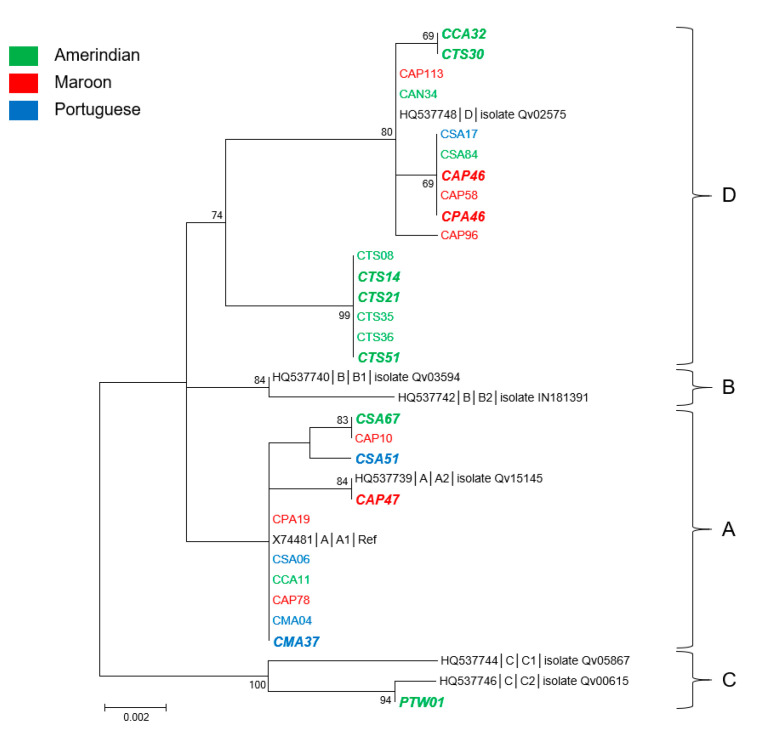
Phylogenetic tree of HPV52 variants from French Guiana. Maximum likelihood phylogenetic tree of HPV52 variants based on 741 bp of E6 and E7 concatenated sequences. Four clusters were identified as lineages A, B, C, and D. Only bootstrap values above 70% are represented in the branches. The tree is drawn to scale, with branch lengths measured in the number of substitutions per site. Newly generated sequences, here identified by their IDs, are color-coded according to the mother tongue of the women from whom they were obtained. Oversized sequences in bold and in italics are those obtained from women with an abnormal cytology (either ASCUS, ASC-H, LSIL, HSIL, or glandular anomalies).

Phylogenetically, all but one sequence fell within two of the four described lineages, A and D (Figure 4). The only exception was a sequence identified from an Amerindian woman from Twenke-Taluen, which belonged to lineage C. Of the 12 other sequences, 10 identified from Amerindian women belonged to lineage D. They were distributed into two main highly supported monophyletic subgroups of which one was only composed of strains (six of the seven) identified from women from the “Trois Sauts” village. These last sequences presented from 98.9% to 99.2% of nucleotide identity and from 98.8% to 99.2% of amino acid identity with all other lineage D sequences. The nine strains identified from Maroon women were equally distributed within A and D lineages, whereas four of the five sequences from women of Portuguese language were of the A lineage. The cross tabulations of lineages and river basins failed to reach statistical significance (*p* = 0.4).

Twelve of 27 sequences were identified from women with cytological anomalies. More than half of them (7/12) were from Native American-speaking women and were distributed in lineages A, C, and D but five of them belonged to lineage D of which three to the “Amerindian” subgroup. The three sequences associated with cytological anomalies from Bushinengue-speaking women belonged to lineages A and D. They represented a third of the HPV52 sequences identified from Maroon women. The last sequence from a woman with an abnormal cytology was identified from a Portuguese-speaking woman and was of lineage A.

### 3.5. Genetic Diversity and Phylogeny of HPV58 Sequences

Fifteen HPV58 sequences were characterized from our cohort (Supplementary Table S7). They were identified from women from two villages from the Oyapock and three from the Maroni. Seven women were Native American-speaking from the Oyapock and six were Bushinengue-speaking from the Maroni. The last two were a Brazilian woman from Saint Georges and a Surinamese woman from Papaïchton.

The E6 sequences presented two nucleotide substitutions at positions 78 and 258, one of which was nonsynonymous leading to the D86E mutation (relative to the reference sequence, accession # D90400). The E7 sequences had seven nucleotide substitutions at positions 121, 188, 220, 225, 228, 267, and 279 associated with four amino acid changes (G41R, G63D, T74A, and D76E). Sequence pairwise comparison demonstrated that eight sequences were identical to each other and to the sequence of variant A2 (accession number HQ537752), whereas the seven others were identical to the sequence of variant C (accession number HQ537774). The difference between these two types of variants was 1.2% at the nucleotide level and 2% at the amino acid level. They demonstrated from 98.5% to 100% nucleotide identity and from 97.2% to 100% amino acid identity with the reference sequences.

**Figure 5:**
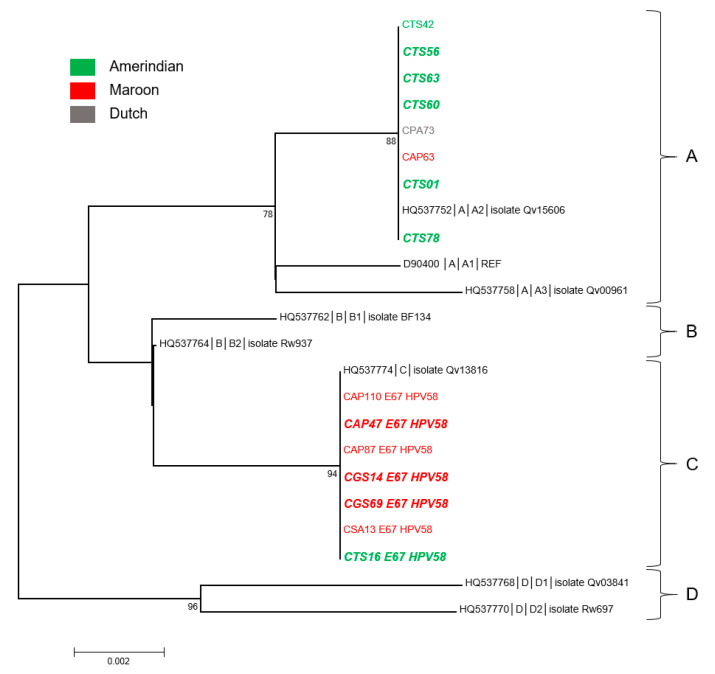
Phylogenetic tree of HPV58 variants from French Guiana. Maximum likelihood phylogenetic tree of HPV58 variants based on 741 bp of E6 and E7 concatenated sequences. Four clusters were identified as lineages A, B, C, and D. Only bootstrap values above 70% are represented in the branches. The tree is drawn to scale with branch lengths measured in the number of substitutions per site. Newly generated sequences, here identified by their IDs, are color-coded according to the mother tongue of the women from whom they were obtained. Oversized sequences in bold and in italics are those obtained from women with an abnormal cytology (either ASCUS, ASC-H, LSIL, HSIL, or glandular anomalies).

As can be observed in Figure 5, six of the eight A2 sequences were identified from Native American-speaking women. The other two were from a Bushinengue-speaking woman and from the Surinamese woman. By contrast, six of the seven C sequences were from Bushinengue-speaking women. The cross tabulations of lineages and river basins suggested patterns but failed to reach statistical significance (*p* = 0.07). Finally, nine of the 15 HPV52 sequences (60%) were identified from women with cytological anomalies (Supplementary Table S7). Remarkably, of the seven sequences from Native American women, six were associated with abnormal cytologies and five were A2 sequences. Half of the sequences (3/6) from Bushinengue women were also associated with abnormal cytologies.

### 3.6. Genetic Diversity and Phylogeny of HPV68 Sequences

HPV68 was the second most prevalent genotype of our study population, with 20 sequences (Supplementary Table S8). As compared with the other genotypes, HPV68 was unevenly distributed with 18 sequences from four villages on the Maroni river and only two from two villages on the Oyapock (Supplementary Table S8). Accordingly, HPV68 was mainly identified from women of Bushinengue language, with 17 sequences identified. The three others were identified from two Native American-speaking women and from one French-speaking woman from the village of Saint Georges.

Over 804 nucleotide positions, the newly identified E6/E7 sequences presented among themselves a total of 59 nucleotide substitutions. The E6 coding sequences presented 39 substitutions of which 15 nonsynonymous associated with amino acid changes at positions 3, 31, 50, 53, 62, 91, 100, 102, 107, 113, 122, 123, 127, 133, and 152, relative to the reference sequence (accession number # DQ080079). The E7 sequences had 20 substitutions associated with 13 amino acid changes (at positions: 18, 22, 33, 52, 64, 68, 74, 75, 85, 89, 91, 92, and 99). Pairwise sequence comparison showed that the new sequences exhibited between themselves as well as with the reference sequences from 94% to 100% identity at the nucleotide level. In addition, they possessed from 90.7% to 100% and from 91% to 100% amino acid identity among them and with the reference sequences, respectively. Nine sequences identified from two villages, Apatou and Papaïchton, were identical to the reference sequence.

**Figure 6:**
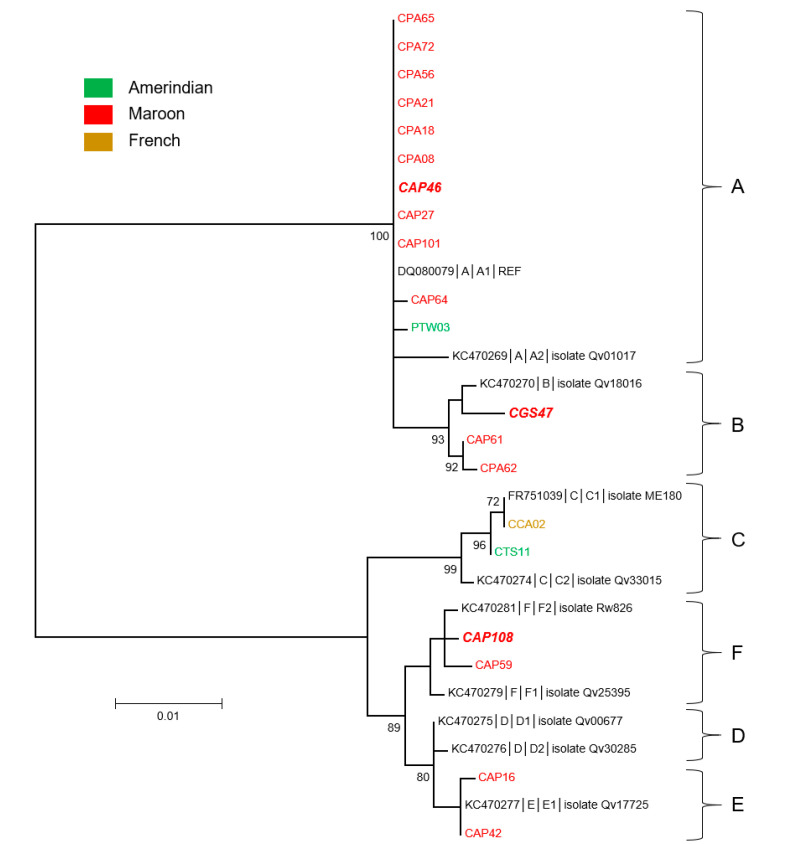
Phylogenetic tree of HPV68 variants from French Guiana. Maximum likelihood phylogenetic tree of HPV68 variants based on 804 bp of E6 and E7 concatenated sequences. Six clusters were identified as lineages A, B, C, D, E and F. Only bootstrap values above 70% are represented in the branches. The tree is drawn to scale with branch lengths measured in the number of substitutions per site. Newly generated sequences, here identified by their IDs, are color-coded according to the mother-tongue of the women from whom they were obtained. Oversized sequences in bold and in italics are those obtained from women with an abnormal cytology (either ASCUS, ASC-H, LSIL, HSIL, or glandular anomalies).

The phylogenetic analysis of these sequences demonstrated that those identified from Bushinengue-speaking women fell within four distinct lineages (A, B, E, and F): 10 belonged to lineage A, 3 to B, and 2 each to both lineage E and F (Figure 6). The only sequence identified from a Native American-speaking woman from the Maroni river was also of lineage A. The last two sequences, identified from women from the Oyapock, even though from women of different origins, were of lineage C. The cross tabulations of lineages and river basins showed strong statistical significance (*p* < 0.001). At last, of the 20 HPV68 sequences, only three were identified in women presenting cytological anomalies (Figure 6 and Supplementary Table S8). These three sequences each belonged to lineages A, B, and F.

## 4. Discussion

The present results show that there were geographically based differences in the genetic sequences of the E6 and E7 oncogenes of the main HR HPV genotypes present in the populations living in the remote villages of French Guiana. The interest of studying these populations is that their ancestors originated from different parts of the world, presumably bringing with them endemic HPVs of their region, and that they have been living in great isolation for generations, with mostly endogamous sexual networks, thus enhancing the likelihood of observing local variations.

Overall, the trees show that there is a geographic clustering of sequences, for which the coarsest level is by river (Maroni vs. Oyapock rivers), which also roughly corresponds to Maroon villages vs. Amerindian villages. There is, however, some heterogeneity between the patterns observed for the different types. The most clear-cut pattern was obtained for HPV18 and HPV58 for which the major branches were crisply divided between Amerindian villages on the Oyapock and Maroon villages on the Maroni. Such clustering was also observed, though not as clearly, for HPV31 and 52. For HPV16, there was also some evidence of clustering on the Oyapock with type A European viruses and on the Maroni with type B and C African viruses among Maroon women. HPV68 had a quite distinct distribution. Indeed, even though it was the second most prevalent genotype of our study population and showed the largest sequence heterogeneity of the six genotypes at both nucleotide and amino acid levels, it was almost exclusively restricted to Maroon women of the Maroni river. The reasons behind this clustering could be the fact that variants came with ancestral populations and remained within the ethnic populations because of endogamy due to cultural traditions and constraints of geography in these remote areas. Generally, it could be observed from the phylogenetic trees that women from the regions requiring a special permit to access (Trois Sauts, Camopi, Antecume Pata) were more homogenous. This observation supports the initial hypothesis that isolated populations would have sustained the ancestral virus populations from their ancestors who arrived from different continents. In contrast, authors in Brazilian cities in the Amazon region, where population is very admixed, could not correlate ancestral origins of women with HPV16 variants [21]. In addition, for both HPV31 and HPV52, we identified subsets of sequences from Native American-speaking women that presented about 0.5% to 1% nucleotide sequence difference with their closest relatives of lineages C and D, respectively. Although, based on E6/E7 sequences, our data do not meet the minimum criteria for defining variants, these sequences could correspond to new HPV31 and HPV52 sublineages specific to this population [22]. Complete genomic sequences of these variants will need to be generated for further characterization.

Among the women included, there were also women originating from different countries, Brazil, Suriname, Dominican Republic, and Guyana, and their viruses branched among those isolated among Maroon and Amerindian women. The gold-rich soil in the interior of French Guiana attracts numerous illegal gold miners, or garimpeiros, and activities that surround gold mining, among which sex work is a frequent corollary [4,23]. In addition, welfare given to local population partly fuels a flourishing sex work market in remote areas where poverty is rife [24]. The nationalities observed in our samples were consistent with the most frequent nationalities of sex workers in French Guiana, suggesting that at least some of these women were engaged in sex work and were consequently infected by the same viruses as those circulating in the community where they worked [25]. Hence, the viruses isolated among these women branched with the lineages from the villages where they were sampled. It is not uncommon for sex workers to rotate between sites to increase their activity with novelty-seeking customers [26]. The specialization in remote sites on the Maroni and Oyapock, on gold mining sites, may bridge the distance between the two river basins and the different relatively endogamous communities, thus leading to singularities in the observed phylogenetic patterns.

The study limitations were that the overall sample size, broken down into subsamples of positives for different genotypes, did not allow sufficient power to clearly test the above bridging hypothesis through sex work. The information about sex work was not available and could only be suspected from past surveys on sexual practices in the region. The cross tabulation between cytological anomalies and different branchings lacked statistical power to detect any clinical differences. Nevertheless, the present study is unique in that it shows populations of different ancestral origins having lived in great isolation until now with endogamous cultural preferences.

## 5. Conclusions

In conclusion, there were geographically based differences in the genetic sequences of the E6 and E7 oncogenes of the main HR HPV genotypes present in the populations living in the remote villages of French Guiana. The geographic clustering of sequences also roughly corresponded to Maroon villages vs. Amerindian villages. The most clear-cut pattern was obtained for HPV18 and HPV58 for which the major branches were crisply divided between Amerindian villages on the Oyapock and Maroon villages on the Maroni.

## Figures and Tables

**Table 1:** HPV genotypes identified on the Oyapock, classified by villages.

						Genotypes					
	*ID*	*Age*	*Country of Birth*	*Monther Tongue*	*Cytology*	*16*	*18*	*31*	*52*	*58*	*68*
**Saint Georges (8 seqs)**	CSA89	43	Brazil	Portuguese			⚫				
	CSA77	32	Brazil	Portuguese				⚫			
	CSA06	26	Brazil	Portuguese					⚫		
	CSA17	26	French Guiana	Portuguese					⚫		
	CSA51	30	Brazil	Portuguese	ASCUS				⚫		
	CSA67	42	French Guiana	Amerindian	HSIL				⚫		
	CSA84	29	French Guiana	Amerindian					⚫		
	CSA13	23	Brazil	Portuguese						⚫	
**Camopi (9 seqs)**	CCA03	48	Brazil	Portuguese	ASCUS	⚫					
	CCA35	28	French Guiana	Amerindian	LSIL	⚫					
	CCA39	35	French Guiana	Amerindian	ASCUS	⚫					
	CCA54	31	French Guiana	Amerindian			⚫				
	CCA25	32	French Guiana	Amerindian				⚫			
	CCA52	56	French Guiana	Amerindian				⚫			
	CCA11	42	French Guiana	Amerindian					⚫		
	CCA32	41	French Guiana	Amerindian	LSIL				⚫		
	CCA02	22	French Guiana	French							⚫
**Trois Sauts (30 seqs)**	CTS23	25	French Guiana	Amerindian		⚫					
	CTS30	26	French Guiana	Amerindian	LSIL	⚫			⚫		
	CTS33	26	French Guiana	Amerindian		⚫					
	CTS43	39	French Guiana	Amerindian	ASC-H	⚫					
	CTS07	30	French Guiana	Amerindian			⚫				
	CTS21	33	French Guiana	Amerindian	ASCUS		⚫		⚫		
	CTS24	31	French Guiana	Amerindian			⚫	⚫			
	CTS29	33	French Guiana	Amerindian			⚫				
	CTS56	37	French Guiana	Amerindian	LSIL		⚫			⚫	
	CTS05	28	French Guiana	Amerindian	HSIL			⚫			
	CTS20	52	French Guiana	Amerindian				⚫			
	CTS37	29	French Guiana	Amerindian				⚫			
	CTS51	38	French Guiana	Amerindian	ASC-H			⚫	⚫		
	CTS52	29	French Guiana	Amerindian				⚫			
	CTS08	22	French Guiana	Amerindian					⚫		
	CTS14	33	French Guiana	Amerindian	LSIL				⚫		
	CTS35	34	French Guiana	Amerindian					⚫		
	CTS36	53	French Guiana	Amerindian					⚫		
	CTS01	35	French Guiana	Amerindian	ASCUS					⚫	
	CTS16	35	French Guiana	Amerindian	LSIL					⚫	
	CTS42	34	French Guiana	Amerindian						⚫	
	CTS60	22	French Guiana	Amerindian	ASCUS					⚫	
	CTS63	32	French Guiana	Amerindian	ASCUS					⚫	
	CTS78	29	French Guiana	Amerindian	ASC-H					⚫	
	CTS61	20	French Guiana	Amerindian							
	CTS11	25	French Guiana	Amerindian							⚫
					Total/genotype	7	7	9	14	8	2

The number of sequences obtained for each village is indicated below the name of the villages. The isolate from which no amplification product has been obtained is identified in light-grey. In the “Cytology” column, only isolates with an abnormal cytology are indicated. ASCUS stands for Atypical Squamous Cells of Unknown Significance; LSIL for Low grade Squamous Intraepithelial Lesion; ASC-H for Atypical Squamous Cell evocating High grade lesion; HSIL for High grade Squamous Intraepithelial Lesion. The dots represent, for each sample, the different genotypes for which sequences have been obtained. The black squares represent the expected genotype based on the Greiner Bio-One screening that has not been amplified. At the bottom of the table is indicated the total number of sequences obtained per genotype.

**Table 2:** HPV genotypes identified on the Maroni, classified by villages.

						Genotypes					
	*ID*	*Age*	*Country of Birth*	*Monther Tongue*	*Cytology*	*16*	*18*	*31*	*52*	*58*	*68*
**Apatou (27 seqs)**	CAP31	34	Suriname	Bushinengue		⚫					
	CAP41	37	Suriname	Bushinengue		⚫					
	CAP11	46	Suriname	Bushinengue	HSIL		⚫				
	CAP87	29	Suriname	Bushinengue			⚫			⚫	
	CAP88	50	French Guiana	Bushinengue			⚫				
	CAP100	26	French Guiana	Bushinengue	LSIL		⚫				
	CAP46	20	French Guiana	Bushinengue	ASCUS			⚫	⚫		⚫
	CAP10	49	Suriname	Bushinengue					⚫		
	CAP47	55	Suriname	Bushinengue	HSIL & GA				⚫	⚫	
	CAP58	60	French Guiana	Bushinengue					⚫		
	CAP78	52	French Guiana	Bushinengue					⚫		
	CAP96	22	French Guiana	Bushinengue					⚫		
	CAP113	26	French Guiana	Bushinengue					⚫		
	CAP63	38	French Guiana	Bushinengue						⚫	
	CAP110	58	Suriname	Bushinengue						⚫	
	CAP57	22	French Guiana	Bushinengue							
	CAP16	51	Suriname	Bushinengue							⚫
	CAP27	64	French Guiana	Bushinengue							⚫
	CAP42	54	French Guiana	Bushinengue							⚫
	CAP59	60	French Guiana	Bushinengue							⚫
	CAP61	48	French Guiana	Bushinengue							⚫
	CAP64	39	Suriname	Bushinengue							⚫
	CAP101	26	Suriname	Bushinengue							⚫
	CAP108	22	French Guiana	Bushinengue	ASCUS						⚫
**Grand Santi (8 seqs)**	CGS04	37	Suriname	Bushinengue		⚫					
	CGS57	23	French Guiana	Bushinengue		⚫					
	CGS64	34	French Guiana	Bushinengue		⚫					
	CGS26	24	Suriname	Bushinengue				⚫			
	CGS31	56	Suriname	Bushinengue				⚫			
	CGS14	26	Suriname	Bushinengue	ASCUS					⚫	
	CGS69	43	Suriname	Bushinengue	ASC-H					⚫	
	CGS47	28	Suriname	Bushinengue	HSIL						⚫
**Papaïchton (19 seqs)**	CPA25	38	French Guiana	Amerindian		⚫					
	CPA79	28	Brazil	Portuguese	ASCUS	⚫					
	CPA45	55	French Guiana	Bushinengue			⚫				
	CPA52	26	Dominican Rep.	Spanish	ASCUS		⚫				
	CPA69	45	French Guiana	Bushinengue			⚫				
	CPA80	50	French Guiana	Bushinengue			⚫	⚫			
	CPA63	39	Dominican Rep.	Spanish	LSIL			⚫			
	CPA73	47	Suriname	Dutch				⚫		⚫	
	CPA19	44	French Guiana	Bushinengue					⚫		
	CPA46	58	French Guiana	Bushinengue	ASCUS				⚫		
	CPA08	42	French Guiana	Bushinengue							⚫
	CPA18	40	French Guiana	Bushinengue							⚫
	CPA21	30	French Guiana	Bushinengue							⚫
	CPA56	61	Suriname	Bushinengue							⚫
	CPA62	37	French Guiana	Bushinengue							⚫
	CPA65	49	Suriname	Bushinengue							⚫
	CPA72	64	French Guiana	Bushinengue							⚫
	CPA01	33	French Guiana	Bushinengue							
**Maripasoula (8 seqs)**	CMA04	26	Brazil	Portuguese	ASC-H	⚫		⚫	⚫		
	CMA10	29	Brazil	Portuguese		⚫					
	CMA52	44	Brazil	Portuguese	ASC-H						
	CMA06	23	Brazil	Portuguese				⚫			
	CMA28	29	French Guiana	Guianese Creole				⚫			
	CMA33	32	Guyana	English	LSIL			⚫			
	CMA37	39	Brazil	Portuguese	ASC-H				⚫		
**Antecum Pata (3 seqs)**	PAN07	55	Brazil	Amerindian	LSIL	⚫					
	PAN12	64	French Guiana	Amerindian		⚫					
	CAN34	35	French Guiana	Amerindian					⚫		
**Twenke-Taluen** **(2 seqs)**	PTW01	30	Suriname	Amerindian	HSIL				⚫		
	CTW27	29	Suriname	Amerindian							
	PTW03	40	Suriname	Amerindian							⚫
					Total/genotype	11	8	10	13	7	18

The number of sequences obtained for each village is indicated below the name of the villages. The four isolates from which no amplification product has been obtained are identified in light-grey. In the “Cytology” column, only isolates with an abnormal cytology are indicated. ASCUS stands for Atypical Squamous Cells of Unknown Significance; LSIL for Low grade Squamous Intraepithelial Lesion; ASC-H for Atypical Squamous Cell evocating High grade lesion; HSIL for High grade Squamous Intraepithelial Lesion and GA for Glandular Anomaly. The dots represent, for each sample, the different genotypes for which sequences have been obtained. The black squares represent the expected genotypes based on the Greiner Bio-One screening that have not been amplified. At the bottom of the table is indicated the total number of sequences obtained per genotype.

## Supplementary Materials

The following are available online at https://www.mdpi.com/2076-2607/8/11/1842/s1, Table S1: Oligonucleotide primers used for E6-E7 PCR amplifications, Table S2: List of isolates used as a reference library for phylogenetic analyses representative of each HPV type, lineage and sub-lineage with their isolate name, accession number and geographical origin, Table S3: HPV16 sequences, classified by villages, Table S4: HPV18 sequences, classified by villages, Table S5: HPV31 sequences, classified by villages, Table S6: HPV52 sequences, classified by villages, Table S7: HPV58 sequences, classified by villages, Table S8: HPV68 sequences, classified by villages.
